# Amyloid precursor protein elevates fusion of promyelocytic leukemia nuclear bodies in human hippocampal areas with high plaque load

**DOI:** 10.1186/s40478-021-01174-x

**Published:** 2021-04-13

**Authors:** David Marks, Natalie Heinen, Lisa Bachmann, Sophia Meermeyer, Michelle Werner, Lucia Gallego, Peter Hemmerich, Verian Bader, Konstanze F. Winklhofer, Elisabeth Schröder, Shirley K. Knauer, Thorsten Müller

**Affiliations:** 1 Department of Molecular Biochemistry, Cell Signalling, Faculty of Chemistry and Biochemistry, Ruhr University Bochum, Bochum, Germany; 2 Institute of Psychiatric Phenomics and Genomics (IPPG), University Hospital, LMU Munich, Munich, Germany; 3 Leibniz Institute On Aging Research, Jena, Germany; 4 Department of Molecular Cell Biology, Institute of Biochemistry and Pathobiochemistry, Medical Faculty, Ruhr-University Bochum, Bochum, Germany; 5 Department of Molecular Biology II, Center of Medical Biotechnology (ZMB), University of Duisburg-Essen, Essen, Germany

**Keywords:** Alzheimer’s disease, APP-CT50, PML, IPSC-derived cerebral organoids, 3D culture, Nuclear complexes, Human brain, Amyloidogenic plaques, HSV, Viral defence

## Abstract

The amyloid precursor protein (APP) is a type I transmembrane protein with unknown physiological function but potential impact in neurodegeneration. The current study demonstrates that APP signals to the nucleus causing the generation of aggregates consisting of its adapter protein FE65, the histone acetyltransferase TIP60 and the tumour suppressor proteins p53 and PML. APP C-terminal (APP-CT50) complexes co-localize and co-precipitate with p53 and PML. The PML nuclear body generation is induced and fusion occurs over time depending on APP signalling and STED imaging revealed active gene expression within the complex. We further show that the nuclear aggregates of APP-CT50 fragments together with PML and FE65 are present in the aged human brain but not in cerebral organoids differentiated from iPS cells. Notably, human Alzheimer’s disease brains reveal a highly significant reduction of these nuclear aggregates in areas with high plaque load compared to plaque-free areas of the same individual. Based on these results we conclude that APP-CT50 signalling to the nucleus takes place in the aged human brain and is involved in the pathophysiology of AD.

**Supplementary Information:**

The online version contains supplementary material available at 10.1186/s40478-021-01174-x.

## Introduction

Increased amyloidogenic processing of the amyloid precursor protein (APP) occurs in sporadic Alzheimer’s disease (AD) [1], in familial AD with mutations in APP or in its processing enzymes [2], and in trisomy 21 patients [3]. Amyloidogenic APP processing causes the generation of three fragments: (i) the secreted extracellular domain (sAPPβ), (ii) the β-amyloid peptide (Aβ), and (iii) the APP C-terminal fragment (APP-CT). The secreted fragment (i) was reported to provoke neurotrophic effects [4], Aβ (ii) is the main component of amyloidogenic plaques [5] and APP-CT (iii) was suggested to play an important role in a nuclear signal transduction pathway [6]. APP-CT, which has been reported to exist in different isoforms with 50aa in length being the most stable one (APP-CT50), indeed is a remarkable protein fragment as it is intrinsically unstructured [7]. Though, this changes upon interaction with FE65, causing APP-CT50 to fold into a three-dimensional conformation that can be analysed by x-ray crystallography. APP-CT50 is capable to enter the nucleus establishing a protein complex consisting of additional proteins like FE65, TIP60, and BLM [8]. The presence of the histone acetyl transferase (TIP60) and the DNA helicase (BLM) in the complex points to a functional role in essential biological mechanisms such as gene expression, DNA replication/damage/repair or chromatin modification. Indeed, a variety of target genes like GSK3β, IDE, and APP have been proposed to be APP-CT50 dependently regulated [9–12].

Promyelocytic leukemia nuclear bodies (PML-NBs) are multiprotein complexes with PML as the main building component [13]. A diverse set of nuclear proteins have been identified as permanent or transient PML-NB-binding partners [14]. PML-NBs are highly dynamic structures with respect to mobility, composition, architecture, and function [15]. While their precise biochemical functions have not been elucidated yet, they have been linked to many aspects of chromatin biology, including transcription, histone modification, repair and recombination, degradation, hence genome maintenance [16]. Transcription of PML is strongly upregulated by interferons and p53 [17], causing a significant increase in the number and size of the bodies. Recent studies revealed an emerging role of PML-NBs as coregulatory structures of both type I and type II interferon responses [18]. Within this work, we demonstrate that PML nuclear bodies interact with highly mobile APP-CT complexes and progressively form immobile large nuclear structures with relevance for AD pathophysiology.

## Methods

### Vector constructs

The plasmids encoding the fusion proteins (FE65-EGFP, FE65-mCherry, APP-CT-GFP, TIP60-EGFP, TIP60-HA, TIP60-BMP, EGFP-PML, PML-HA, PML-myc, p53-EGFP, Daxx-EGFP, H2A-mTurquoise, HIPK2-EGFP, HP1ß-EGFP, UBE2D2-mCherry, and WRN-EGFP) described in this paper, were generated using the In-Fusion® HD cloning kit (Takara Bio) according to manufacturer’s instructions or were purchased (Addgene). Amplification and purification of the plasmids were done according to standard protocols. An overview of all constructs used in this study is given in Additional file 1: Fig. S7.

### Cell culture, transfection, and immunofluorescence

Stem cells (iPS CD34) were cultured in StemFlex™ (Gibco) on 35 mm dishes, coated with Matrigel or Geltrex® (Gibco) according to the manufacturer’s protocol, and split before reaching 70% confluency. HEK293T cells were seeded and incubated in DMEM (Gibco) with 10% heat inactivated FBS (Gibco), 1% Penicillin/Streptomycin and 1% L-glutamine (Gibco), to a confluency of 70%. For overexpression assays, sterile precision cover glasses (1.5 H Marienfeld Superior) were placed into a 24-well cell culture plate (Sarstedt) and coated with 0.01% poly-l-ornithine solution (Sigma Aldrich). The respective plasmids were transfected via the K4® Transfection Kit (Biontex) according to manufacturer’s recommendations for 24-well culture plates. After 24 or 48 h, cells were briefly washed with DPBS (Gibco) and fixed in 4% paraformaldehyde in PBS. Cover glasses were mounted with the Shandon™ Immu-Mount™ solution (Thermo Scientific) on glass slides and dried overnight at RT. For immunofluorescence staining, HEK293T cells were seeded in 8-well-µ-slide-ibi Treat (ibidi®, Martinsried, Germany) and transfected using calcium phosphate transfection after 24 h. Cells were fixed with Roti®-Histofix 4% (4% phosphate buffered formaldehyde solution; Roth, Karlsruhe, DE) for 20 min at 37 °C, and permeabilized and blocked with 5% normal goat serum (NGS) in 0.3% (w/v) Triton X-100/PBS for 30 min at RT. The cells were incubated with primary antibodies diluted in 1% BSA/0.3% Triton X-100/DPBS (mouse anti-HA (BioLegend, 901501; 1:1000), mouse anti-myc (NEB/Cell Signalling, 2276; 1:1500) o/n at 4 °C. For the secondary antibody staining and the cell staining, goat-anti-mouse AF568 (Invitrogen, A11004, 1:1000) was used, together with HCS CellMask™ Deep Red Stain (ThermoFisher Scientific, H32721, 1:5000), Hoechst33342 (10 mg/mL in H2O, Applichem, A0741, 1:1000) in DPBS (1% BSA, 0.3% Triton) and incubated for 1 h at RT.

#### Immunoprecipitation

HEK 293 T cells were seeded in 10 cm dishes and co-transfection was performed 24 h after seeding. Whole cell extracts were prepared 24 h after transfection by scraping the cells from the dish with a cell scraper, washing the cell pellet in ice-cold PBS, extracting with 1 ml interaction buffer (50 mM Tris pH 8, 150 mM NaCl, 5 mM EDTA, 0.5% NP40, 1 mM DTT, 1 mM PMSF, 1× complete protease inhibitor cocktail), followed by sonication (15 s at 95% amplitude) using a Sonopuls mini20 device (Bandelin, Berlin, Germany). The lysates were centrifuged (15,000*g*, 15 min, 4 °C) and the supernatant was transferred to a new reaction tube. Input samples of the lysates were stored separately. Immunoprecipitation (IP) was carried out with the µMACS isolation kits for tagged proteins from Miltenyi Biotec (Bergisch-Gladbach, Germany). The eluates, as well as input samples of the lysates were subjected to SDS-PAGE and immunoblotting.

#### Immunoblotting

Protein concentrations were determined using the Bio-Rad protein assay system (Bio-Rad Laboratories, Richmond, CA). Equal amounts of protein were resolved by SDS-PAGE using a 10% acrylamide gel and subsequently transferred onto polyvinylidene difluoride (PVDF) membranes (Amersham Hybond, GE Healthcare) via the PerfectBlue™ tank electro blotter (Peqlab, Erlangen, Germany) with 350 mA for 90 min. To minimize unspecific binding, the membranes were blocked in 5% (w/v) non-fat dried milk powder in TBST for 30 min at RT. Membranes were probed with primary antibodies against GFP (rabbit, polyclonal, 1:2000, Santa Cruz, sc-8334), HA tag (mouse, monoclonal, 1:1000, BioLegend, 901501) and p53 (mouse, monoclonal, 1:200, Novus Biologicals, NBP2-29419) diluted in blocking solution overnight at 4 °C. The membranes were washed three times with TBST, before they were incubated with HRP-conjugated secondary antibodies (1:10,000, NXA931 and NA934, GE Healthcare Europe, Freiburg, DE) also diluted in blocking solution for 1 h at RT. Visualization of bound antibodies occurred via enhanced chemiluminescence (ECL) with the ECLplus Western Blotting Substrate from Pierce (Rockford, IL, USA) according to the manufacturer’s instructions. After incubation with the substrate, the detection of the generated signal was carried out with the ChemiDoc MP Imaging System (Bio-Rad Laboratories GmbH, Feldkirchen, Germany).

### Cerebral organoids

Cerebral organoids were generated according to the protocol from Lancaster and Knoblich [19] with minor modifications, all media compositions remained unchanged. Briefly, at day 0, iPS CD34 positive cells were detached and harvested using TrypLE™ (ThermoFisher, Germany). Afterwards, DMEM/F12 was added to the detached cells and the cell number was calculated using a Neubauer chamber. Next, 9000 cells/well were seeded into a 96-well ultra-low attachment plate (Corning) with a total amount of 150 µL hESC-medium (containing 4 ng/mL bFGF and 50 µM ROCK-Inhibitor) per well. On day 3, half of the media was exchanged with 150 µL of hESC-medium without bFGF and ROCK-Inhibitor. Subsequently (day 6), the embryoid bodies were transferred to a 24-well ultra-low attachment plate (Corning) with 500 µL Neural Induction (NI) -medium. Every day, half of the media was exchanged with 500 µL fresh NI-medium. On day 12, the embryoid bodies were embedded in droplets of Matrigel (Corning) and incubated for 25 min at 37 °C for Matrigel polymerization. Afterwards, the droplets were transferred to a 50 mm dish with differentiation medium without vitamin A (DM-A) medium for further incubation at 37 °C in a 5% CO_2_ atmosphere. Four days later, the medium was changed to DM+A and the developing cerebral organoids (COs) were maintained at 37 °C with 5% CO_2_ until experiments were performed.

#### Histology and immunohistochemistry

The COs were removed from the media, washed with PBS, and fixed with 4% paraformaldehyde in PBS for 90 min at 4 °C. After washing with PBS, organoids were incubated in 30% sucrose solution for cryoprotection at 4 °C overnight. The next day, the COs were embedded in a 1:1 mixture of 30% sucrose and Tissue-Tek O.C.T. embedding medium (Science Services, SA62550-01), snap-frozen on dry-ice, and then stored at − 80 °C until cryosectioning. Frozen COs were sliced into 15 µm sections using a cryostat (Leica CM3050S), mounted on SuperFrost™ slides (ThermoScientific™), and stored at − 80 °C until further use.

For immunohistochemistry, COs and brain tissue sections were thawed for 2 min in PBS. To apply the biotin-avidin system used for the enhancement of fluorescence, the sections were first blocked with avidin for 10 min and, after washing twice with PBS for 4 min, blocked with biotin for 10 min. After washing twice with PBS for 4 min, sections were blocked and permeabilized in 0.1% Triton X-100, 5% goat serum in PBS for 1 h at RT, followed by incubation with primary antibodies in a humidified chamber overnight at 4 °C. Primary antibodies were diluted in 0.1% Triton X-100 in PBS as follows: APP-CT (mouse, Millipore MAB343, 1:100), PML (rabbit, Novus Biologicals NB100-59787, 1:400), PML (mouse, Abcam ab96051, 1:200), TIP60 (mouse, Abcam Ab54277, 1:400), FE65 (mouse, Acris AM32556SU-N, 1:400), FE65 (rabbit, Santa Cruz sc-33155, 1:400), β-tubulin III (mouse, StemCell 01409, 1:100), p53 (mouse, Novus Biologicals NB200-103, 1:100). Sections were incubated with the biotinylated secondary antibody (goat anti-mouse IgG Biotin, Life Technologies B-2763, 1:100) diluted in 0.1% Triton X-100 in PBS for 1 h at RT in a humidified chamber. Following washing twice with PBS for 4 min, sections were incubated with Avidin-TRITC (1:1000) and a non-biotinylated secondary antibody (donkey anti-rabbit FITC, Santa Cruz sc-2090, 1:100) diluted in 0.1% Triton X-100 in PBS for 45 min in a humidified chamber protected from light at RT, and subsequently washed twice with PBS for 4 min.

In case of thioflavin-S counterstaining of amyloid plaques, sections were incubated with 0.1% aqueous thioflavin-S solution, washed twice with PBS for 4 min, washed with 30% ethanol followed by 50% ethanol for 5 min each, and finally washed twice for 4 min with PBS.

For counterstaining of nuclei, DAPI solution (0.001 mg/mL) was added to the sections for 15 min while protected from light, then slides were washed twice with PBS for 4 min and mounted.

#### Imaging and tracking

Cells were either imaged after fixation and mounting (Shandon™ Immu Mount™ solution, Thermo Scientific) on glass slides or for life cell imaging directly using the integrated incubation chamber of the Leica (Mannheim, Germany) TCS SP8 microscope system (37 °C and 5% CO_2_). Samples were imaged using a 63× water (1.2 NA) or 100× oil objective (1.4 NA). Fluorophores were excited with 405/488/514/561 nm laser lines performing a sequential scan beginning with the most red-shifted wavelength. Images were recorded into 1024 × 1024 images at a scan speed of 200 Hz with HyD detectors. Tile scans were imaged through the selection of 800 × 800 µm areas (5 × 5 tiles) in x- and y-direction. Additionally, z-stacks (n = 5) of 2 µm between each plane (8 µm in total) were recorded and merged via the maximum projection tool in the LASX-software (Leica, Mannheim, Germany). The fluorescence intensity curves were measured along the cell nucleus within a region of interest (ROI) and the chromatogram was normalized using the quantitative tools of the LASX-software tool (Leica, Mannheim, Germany). For the 3-dimensional imaging, several z-stacks (n = 10) of 1 µm step size were recorded and the 3-dimensional image was generated using the LASX-software tool.

The track analyser of the Hyugens object tracker wizard was used to study the 3-dimensional motion of the nuclear bodies of cells that were previously transfected with and without PML. Therefore, ROIs containing nuclear bodies only or background only were selected for the tuning of the detection filters via linear discrimination analysis (LDA) and the subsequently tracking of the nuclear bodies. The detection threshold was adjusted to measure objects with a positive generated score, computed by the software, to further discriminate against the background. The bodies were tracked within the cells over a time span of 300 s and the speed was calculated using the integrated software.

#### STED microscopy

For STED, GFP fusion proteins in fixed cells were labelled with Alexa Fluor 647-coupled GFP nanobodies (GFP-booster gb2AF647-50, Chromotek, Germany) at 1:100 dilution. Endogenous and overexpressed PML was immunofluorescently labelled with anti-PML antibody (rabbit, ABD-030, Jena Bioscience, Germany, 1:500), followed by secondary antibody coupled with STAR 580 STED dye (goat-anti-rabbit, ST580-1002-500UG, Abberior, Göttingen, Germany, 1:100). Stained cells were embedded in ProlongGold with DAPI (Thermo Fisher Scientific, Germany) and covered with 12 mm round cover glasses (Thickness 0.17 ± 0.01 mm). Gated STED images were acquired on a Leica TCS SP8 STED microscope equipped with a 100× oil objective (HC PL APO CS2 100×/1.40 Oil) according to protocols established for nuclear bodies by Okada and Nakagawa [20]. Pixel size in STED acquisition was applied automatically in LAS-X software (Leica, Mannheim, Germany) for the most red-shifted dye (AF 647), usually resulting in a pixel size of less than 20 × 20 nm. STED beam alignment was performed before each imaging session between the pulsed white light laser and the 592 nm depletion laser. DAPI, Alexa Fluor 488, Star 580 and Alexa Fluor 647 were excited with laser lines 405 nm, 488 nm, 580 nm and 635 nm of the white light laser, respectively. Emission was captured through band pass settings 430–470 nm, 505–550 nm, 590–620 nm and 648–720 nm, respectively. Depletion of STAR 580 and AF 647 was performed with the 775 nm depletion laser. The power of the depletion laser was optimized for each dye to obtain highest resolution while minimizing bleaching. Imaging conditions were fine-tuned on several cells before application of the optimized settings for final images. Each dye was imaged in sequential scans to avoid spectral overlaps. While hybrid detector gain was set to 100%, excitation laser intensity was set such to prevent pixel saturation. Images were obtained using a pixel dwell time of 100 ns. Photon time gating was employed by collecting lifetimes between 0.3 and 6.0 ns. To compensate for inevitable signal intensity loss during STED acquisition, the excitation laser power was set three–fivefold higher than in conventional confocal mode. When using STED channels, the pinhole was set 1.0 Airy Units. In non-STED channels the pinhole was set to 0.49 Airy Units to allow for sub-Airy super-resolution confocal microscopy according to the HyVolution II mode of the Leica SP8 microscope system. All images were deconvolved with Huygens Professional Software (Scientific Volume Imaging B.V., Hilversum, The Netherlands) using the deconvolution pre-settings in Huygens software applying Classic Maximum Likelihood Estimation (CMLE) algorithms.

#### Cell profiler data analysis

Brain tissue slides acquired from patients with Alzheimer’s disease in different stages of severity, were stained with Thioflavin, DAPI and anti-PML antibodies (as described before). The slides were imaged using the Leica TCS SP8 confocal microscope system with a 100× oil objective (HC PL APO CS2 100×/1.40 Oil) and tile scans (10 × 10 tiles) containing amyloidogenic plaques were recorded. Additionally, several plaque-free regions were captured as control (n = 9 from 3 individuals). PML bodies and cell nuclei were identified and counted using the CellProfiler™ Software. In the tile scans, rectangular areas around the plaques were defined as ‘plaque near’ (n = 31 from 8 individuals) and the surrounding area as ‘plaque distant’ (n = 28 from 7 individuals), not including cells close to the edge of the tile scans. The acquired data from CellProfiler™ were exported to Excel, sorted in those two groups and compared regarding the amount of PML bodies inside the cells and the percentage of cells containing those aggregates.

## Results

### APP-CT induces gene expression-active aggregates with donut-like shape in a variety of cells

**Fig. 1:**
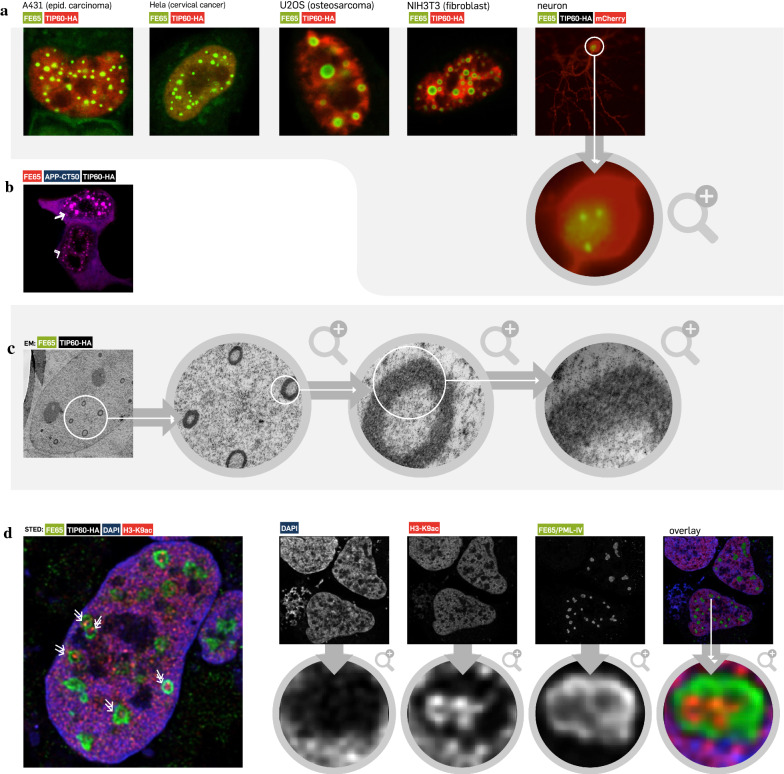
Dynamic nuclear aggregates are present in various cells, lack a membrane coating and are transcriptionally active. (**A**) Upon co-expression of FE65-EGFP (green) and TIP60-HA (w/o fluorophore, stained using anti-HA tag antibody (red)), nuclear aggregates in various sizes are generated in multiple cell lines including neurons (for neurons no co-staining was done as a mCherry vector was additionally co-transfected to identify neuronal cell structure). Transfected vectors with respective fluorophore (EGFP, mCherry) are indicated. (**B**) Every cell type used in **A** demonstrated cells with many tiny (arrow) or few large spheres (arrowhead) (or transition states) supporting the hypothesis of sphere fusion over time (blue, APP-CT50; red, FE65-mCherry, TIP60-HA un-stained; a different overview image is also given in Additional file 1: Fig. S1). (**C**) Transmission electron microscopy of FE65/TIP60-HA transfected cells revealed an electron-dense ring structure (additional image in Figure S2). However, there was no evidence for a membrane sheath. Additionally, these results were also confirmed by a CellMask staining (Additional file 1: Fig. S3). (**D**) High-resolution STED imaging revealed that the inner core of the aggregates is positive for anti K9 acetylation histone 3 antibody staining (red, RFP) supporting the hypothesis of active gene expression within the aggregates

Our study was initiated by studying the presence of the potential nuclear APP signalling pathway in various cell lines including primary neurons (Fig. 1). As published earlier, co-expression of FE65/TIP60 (omitting APP-CT50) is sufficient to establish the nuclear dot-like phenotype [6, 8]. In order to not overwhelm the cell with unnecessary expression constructs, we passed on APP-CT50 expression for some of the subsequent experiments. Indeed, many different cell lines including cancer cells, fibroblasts and primary neurons revealed the typical dot-like phenotype upon co-expression of the complex components (Fig. 1A). All analysed cell types exhibited the same phenotypes—from cells with many tiny aggregates (Fig. 1B, arrow) to those with a few large speckle-like structures (Fig. 1B, arrowhead; an overview image in Additional file 1: Fig. S1). Live cell imaging demonstrated a phenotype of the complexes resembling a highly dynamic circular structure, suggesting being membrane coated aggregates. However, electron microscopy analysis (Fig. 1C; Additional file 1: Fig. S2) as well as CellMask™ membrane stain (Additional file 1: Fig. S3) argued against this hypothesis and rather revealed a donut-like shape of the intranuclear aggregates with an electron-dense border and an electron-poor centre. According to present literature [9–11], a potential function of the APP-dependent nuclear aggregates is the modulation of gene expression in dependence of yet unknown stimuli. In order to test this hypothesis, transfected cells were fixed and stained with an anti-Histone3-K9ac antibody recognizing transcriptional active loci. Indeed, high resolution STED indicated active gene expression (positive staining in red) within large ring-like structures (Fig. 1D, arrows; Additional file 1: Fig. S4).

### The nuclear APP-CT complex associates to two tumour suppressor proteins

**Fig. 2:**
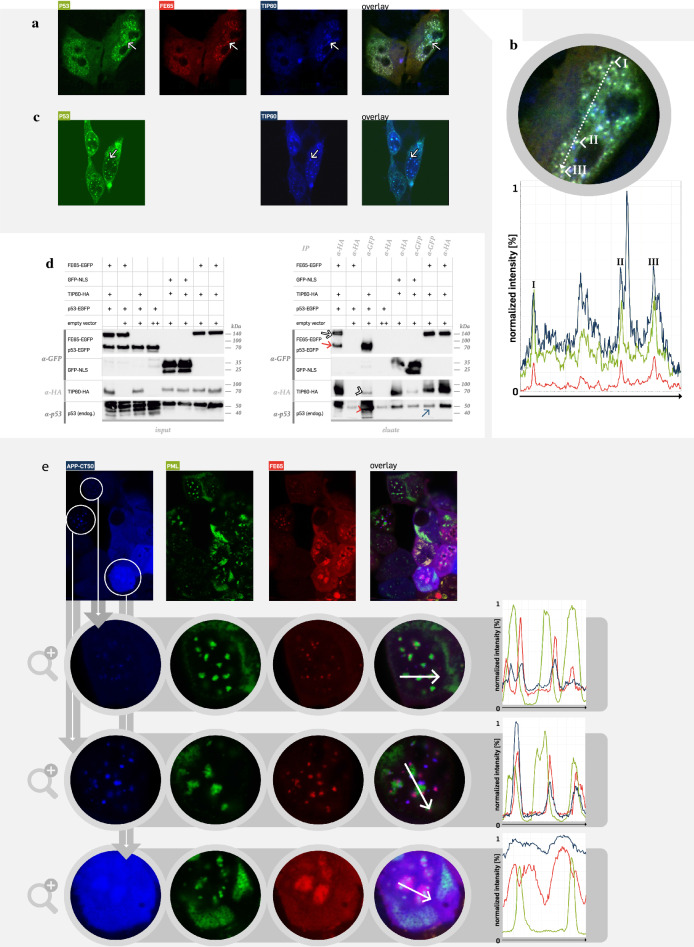
The nuclear APP-CT50 complex associates to p53 and PML. (**A**) FE65 (red, mCherry) and TIP60 (blue, BFP), which are known to co-localize and form a dot-like structure, were transfected into HEK293T cells. With the addition of the tumour suppressor protein p53 (green, EGFP), the same pattern could be revealed (for example see white arrows), proving further co-localization and interactions of the proteins. (**B**) Co-localization of the protein complex is proven by tracking of the fluorescence intensity along the indicated arrow, which revealed peak intensities of each component (green: p53-EGFP, red: FE65-mcherry, blue: TIP60-BFP) supporting the association of all components within one complex. (**C**) Omitting FE65 and transfecting only p53 and TIP60 also revealed a co-localization (for example see white arrows), proving that the complex is independent of FE65. (**D**) P53 interaction with the APP-CT50 complex (FE65-EGFP/ TIP60-HA co-transfected to p53-EGFP versus control (EGFP-NLS, nuclear localization sequence)) was validated by co-immunoprecipitation (left side: input blot, right side: elution blot). IP using anti-HA tag antibody (against TIP60-HA) revealed precipitation of FE65 (white arrow) as well as of p53 (red arrow). Respective controls did not show a co-precipitation. TIP60-HA precipitation also occurred using anti-GFP as bait in well agreement to results obtained in part C (white arrowhead). Notably, high levels of endogenous p53 co-eluted in the same condition (red arrowhead), whereas a moderate endogenous p53 signal was observable in control conditions (red arrow). (**E**) A second tumour suppressor protein was identified to associate with the APP-CT50 complex: the promyelocytic leukemia protein PML. Different phenotypes of association were observed, e.g. one or two APP-CT50 (blue, BFP)/FE65 (red, mCherry) dots (TIP60-HA was co-transfected w/o fluorophore) associated with a single PML (green, EGFP) aggregate (first and second zoom-in row, compare fluorescence intensities). Alternatively, large APP-CT50/FE65 complexes with enclosed PML-dots were found (third row)

Next, we aimed to identify the protein composition of these structures in more detail. Therefore, we extracted a set of proteins from the literature revealing a nuclear phenotype similar to the APP-CT50-dependent aggregates, which resulted in a list of the following proteins: Daxx, H2A, HIPK2, HP1ß, p53, PML, UBE2D2, and WRN. For all of these proteins, vectors encoding the respective candidate DNA sequence fused to a fluorescent protein cassette were cloned and co-transfected with expression constructs for the APP-CT50 complex (APP-CT50/FE65/TIP60). The vast majority of our candidates showed no co-localization with two exceptions. One protein has been described before to be part of nuclear bodies [21] and to bind to BLM [8, 22], as well as to reveal an unstable interaction to TIP60 [23]: the tumour suppressor protein p53. Indeed, co-expression of p53-EGFP, FE65-mCherry and TIP60-BFP demonstrated a strong co-localization in nuclear aggregates (Fig. 2A). This was validated by profiling of the individual fluorescence intensities (Fig. 2B), all three fluorescent signals revealed the same intensity course along the dotted line with peaks at position I, II, and III (Fig. 2B). Omitting FE65 co-expression demonstrated p53 co-localization to TIP60 speckles alone as well (Fig. 2C), which is in line with earlier results [23]. To further confirm the interaction of p53 with APP-CT50-depending complexes, co-immunoprecipitation assays (co-IPs) were performed (Fig. 2D). Sample conditions were selected as indicated in the input blot (Fig. 2D, left). Co-IP (Fig. 2D, right) using anti-HA tag antibody (TIP60-HA) revealed precipitation of FE65 (as expected, Fig. 2D, white arrow) and of p53 (red arrow), which validated its participation in the complex. The TIP60/p53 interaction (independent of FE65, in agreement to Fig. 2C) could be confirmed by precipitating TIP60 via a GFP tag antibody (white arrowhead). In addition to the p53-EGFP signal, the presence of co-precipitated endogenous p53 was observed (3rd land, red arrowhead). Endogenous p53 is also detectable in conditions with TIP60 co-expression (e.g. lane 7, blue arrow) suggesting an interaction of the (endogenous) tumour suppressor protein p53 with the histone acetyltransferase TIP60 in nuclear aggregates.

**Fig. 3:**
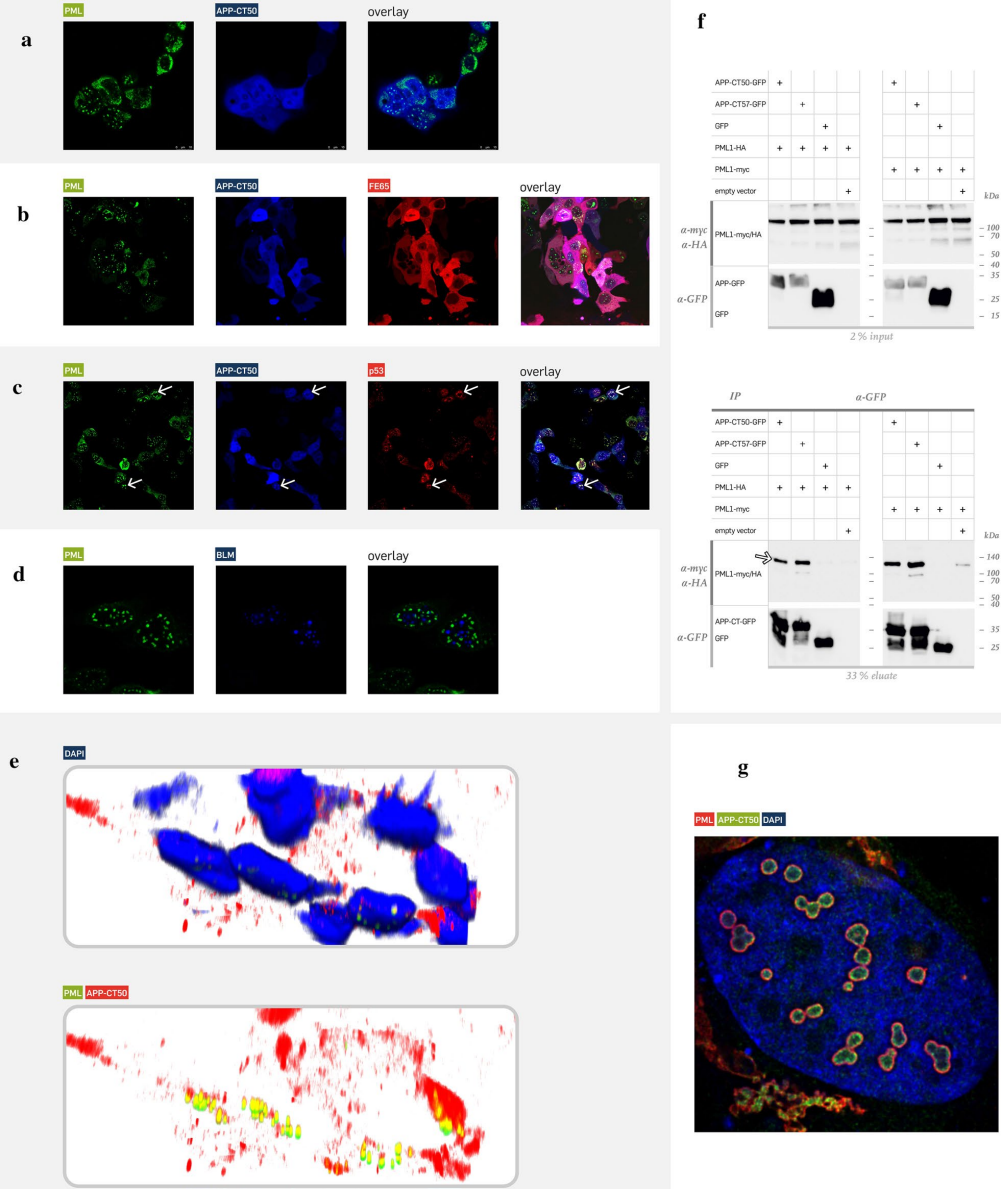
PML forms container-like structures within the complex and precipitates with APP-CT. (**A**) A direct co-localization of APP-CT (blue, BFP) with PML (green, EGFP) was only observed to some extent (cells with nuclear aggregates), but most cells revealed a uniform staining pattern of APP-CT50 and PML in the cytosol and nucleus. (**B**) Additional co-expression of FE65 (red, mcherry) enriched the aggregation of PML in the nucleus, but a strong co-localization to APP-CT50/FE65 was not observable in the imaging study. (**C**) Additional transfections showed that p53 (red, mCherry) is part of the PML aggregates in the nucleus that also contained APP-CT50, as indicated by the visible co-localization (for example see white arrows). (**D**) Notably, another suspected binding partners for PML like the DNA helicase BLM (blue, BFP), which was identified as binding protein in the APP-CT50 complex, is not co-localized with the PML aggregates. (**E**) Confocal 3D imaging validated the co-localization of PML (green, EGFP) and APP-CT50 (red, mCherry) in the nucleus (FE65/TIP60 were co-transfected w/o fluorophore). (**F**) Interaction of APP-CT with PML was shown using co-immunoprecipitation assay. Precipitation using anti-GFP antibody revealed detection of APP-CT-EGFP (as expected) as well as PML (PML1 isoform was used, white arrow). This was true for two different APP-CT isoforms (APP-CT50 and CT57), whereas control conditions revealed no unspecific co-precipitation. The before mentioned isoforms of APP’s c-terminal domain differ in their respective amino acid length generated through ε-cleavage. While APP-CT50 represents the most common form, APP-CT57 is less common.Results were the same for two different PML1 tags: in the left panel of blots PML1-HA was used, whereas PML1-myc was used in the right panel. (**G**) High-resolution STED imaging specified the localization of APP-CT50 (green, EGFP) within the PML bodies (red, mCherry). Different phenotypes were evident, either with a uniform localization within the bodies or with APP-CT50 signal at the inside wall of the PML body

The second candidate was a tumour suppressor protein as well, the promyelocytic leukemia protein (PML) [13]. Co-expression of PML-EGFP, BFP-APP-CT50, FE65-mCherry and TIP60 (w/o fluorophore) revealed a close association of PML bodies to one or two APP-CT50 aggregates (Fig. 2E, zoom 1), which was also confirmed by the fluorescence intensity line scan given in Fig. 2E on the right. Indeed, the green PML peak is accompanied by two peaks of APP-CT50 (blue) and FE65 (red). Other cells of the same condition (zoom 2nd line) demonstrated association of three APP-CT50 complexes, whereas another phenotype (zoom 3rd line) demonstrated large APP-CT50 complexes with incorporated PML bodies. Collectively, the high dynamics of the nuclear complexes was compelling and pointed to a spatial-temporally highly organized mechanism. Direct co-localization of PML with the APP-CT50 fragment (w/o FE65/TIP60 co-expression) was also observed in a minor percentage of transfected cells (not shown), however, the main phenotype revealed a uniform APP-CT50 signal without accumulation in nuclear aggregates (Fig. 3A). Nevertheless, cytosolic PML might also be bound to APP-CT50. Additional expression of FE65 did not change the main phenotype of uniformly localized APP-CT50 (Fig. 3B). Omitting TIP60 co-expression caused the generation of a PML/APP-CT50/p53 complex (Fig. 3C). In contrast, the DNA helicase BLM is not co-localized to PML (Fig. 3D). A more detailed analysis, utilizing 3D confocal imaging, revealed that APP-CT50 and PML are associated with each other in all nuclear aggregates (Fig. 3E). Finally, the PML/APP-CT50 interaction was confirmed by a co-immunoprecipitation assay (Fig. 3F). Co-expression of APP-CT with either PML1-HA or PML1-myc demonstrated precipitation of PML (detection via anti-myc or anti-HA antibody, Fig. 3F, white arrow) upon anti-GFP IP (independent experiment given in Additional file 1: Fig. S5). This was true for two different APP-CT isoforms with 50 and 57 aa in length. High-resolution STED imaging further specified the co-localization of PML and APP-CT50 within the PML bodies. APP-CT50 was either uniformly distributed or concentrated at the inner wall of the nuclear body (Fig. 3G).

### APP-CT50 depending complexes drive PML complex generation that are also present in the aged human brain

**Fig. 4:**
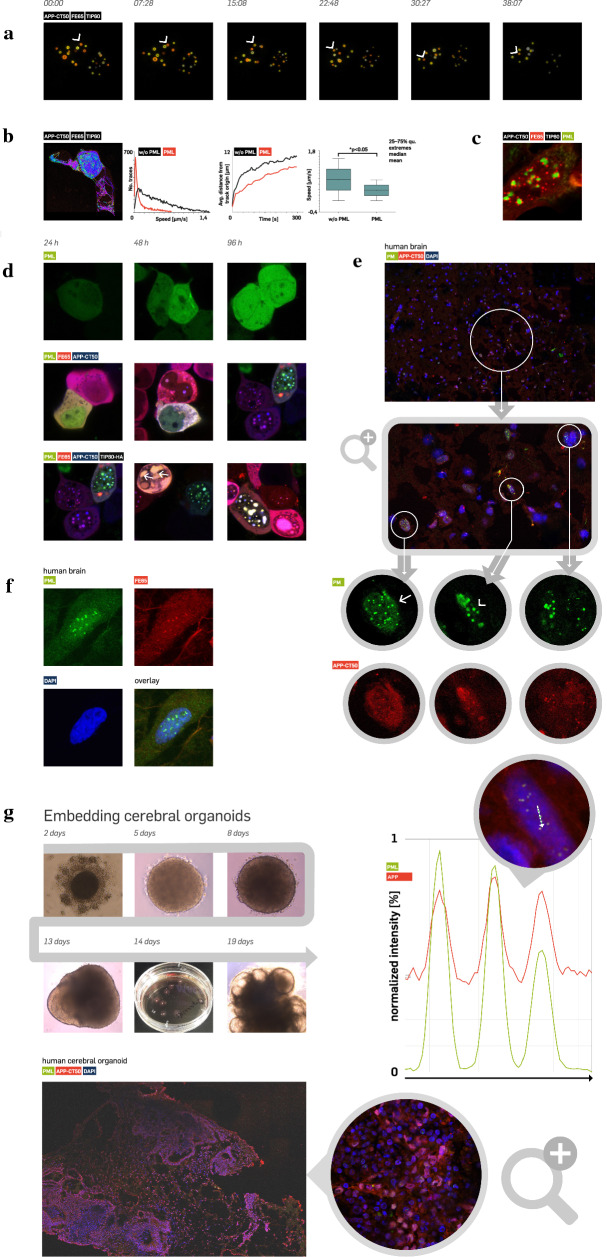
Highly mobile APP-CT50-depending complexes that are also present in the aged human brain drive PML complex generation. (**A**) Expression of APP-CT50/ FE65/TIP60-HA in HEK293 cells reveals a highly mobile complex moving three-dimensionally in the cellular nucleus. Ring-like structures were coloured (orange to yellow) according to z-level (confocal microscopy). The indicated structure (white arrowhead) revealed time-dependent movement. The corresponding video is given in the supplement. (**B**) Movement of the individual aggregates was tracked using Huygens object tracker software. Transfection in HEK293 cells included APP-CT50/FE65-mCherry/TIP60-HA with and without EGFP-PML co-expression. The FE65-mCherry signal was used for tracking, revealing lower speed in cells co-expressing PML (first diagram). In addition, the distance from the track origin (at time point 0) was analysed. Co-expression of PML revealed significantly lower distances pointing to mutual trapping of both complexes. The mean speed was 0.38 in PML versus 0.76 µm/s in non-PML co-expressing cells (last diagram). (**C**) This part reveals a representative image demonstrating the complex generation of APP-CT50/FE65/TIP60 (red) and PML (green aggregates). (**D**) Time-dependent generation of nuclear APP-CT50/PML aggregates. PML (green, EGFP) expression revealed a uniform distribution within the nucleus and cytosol (first row). Co-expression of FE65 (red, mCherry) and APP-CT50 (blue, BFP) caused initial aggregate formation after 48 h (middle row). Additional expression of TIP60-HA (w/o fluorophore) (last row) showed early generation of nuclear aggregates after 24 h. 48 h after transfection large nuclear aggregates were observed (white arrow). (**E**) PML (green, FITC) and APP-CT50 (red, TRITC) co-localization was studied in human brain sections. In total, 15 human hippocampal sections were analysed (different Braak stages). Confocal tile-scan imaging (5 z-stacks, then fused by maximum projection algorithm) revealed strong co-localization of PML with APP-CT50. As in cell culture experiments, nuclei containing many small aggregates (arrow) as well as nuclei with larger aggregates (arrowhead) were evident. Co-localization is further shown by intensity tracking of both fluorescent channels in the diagram for a nucleus along the dotted white arrow. (**F**) Similarly, co-staining of PML (green, FITC) and FE65 (red, TRITC), which confirmed the association of both proteins in the nuclei of the human brain, was performed. (**G**) In order to address the question whether co-localization also occurs in non-aged tissue, we differentiated human cerebral organoids from induced pluripotent stem cells. Embryonic bodies were embedded in Matrigel at day 11 followed by neuronal induction to generate organoids, which were analysed after 30 days in culture (seeding at day 0). Staining of cryosections failed to demonstrate co-localization of APP-CT50 (red, TRITC) and PML (green, FITC)

To further investigate this interaction, live cell imaging experiments were performed in HEK293 cells with ectopic expression of the aggregate components. Expression of FE65-EGFP, TIP60 (w/o fluorophore) revealed highly dynamic ring-like structures moving throughout the nucleoplasm (Fig. 4A, arrowhead, confocal image, structures coloured according to z-level; full video given in the Additional file 2). To investigate the influence of PML in these dynamics, the complexes (based on the FE65-mCherry signal) were tracked in APP-CT50/FE65/TIP60-transfected cells with versus without PML co-expression (Fig. 4B). The velocity of the nuclear structures in the presence of PML (Fig. 4B, diagram 1, red graph) was significantly reduced compared to the condition without PML (black graph). Moreover, the average distance from the track origin was significantly less in PML co-expressing cells (Fig. 4B, diagram 2). The mean speed was 0.38 in PML versus 0.76 µm/s in non-PML co-expressing cells (Fig. 4B, diagram 3). These results suggest a mutual trapping function of APP-CT50 aggregates and PML, for which Fig. 4C shows the typical phenotype of up to three APP-CT50 aggregates (red) bound to a single PML body (green). In order to understand the conditions for aggregation in more detail, we monitored transfected cells over time (Fig. 4D). Pure PML expression (upper row) revealed a homogenous distribution of the protein within the whole cell (cytoplasm and nucleus). Co-transfection of APP-CT50 (blue) and FE65 (red) caused late aggregate formation mostly after 96 h (middle row), whereas expression of PML/FE65/APP-CT50/TIP60 demonstrated nuclear body formation already after 24 h (below row). After 48 h, some cells revealed formation of super-aggregates within the nucleus (white arrow). We subsequently aimed to investigate whether these complexes are present in the human brain as well (Fig. 4E). Human hippocampal frozen brain samples from 15 AD patients were used to study the co-localization of APP-CT50 and PML. We observed a strong co-localization of PML and APP-CT50 in the human brain (Additional file 1: Fig. S6, an antibody raised against APP 643–695 was used in order to identify APP-CT50 in the nucleus). Tracking of the fluorescence intensity along the white arrow (including three aggregates) validated the strong co-localization. We also investigated the localization of FE65 in the same manner (Fig. 4F) and demonstrated that FE65 co-localized with PML in the human brain as well. Both co-localizations were evident in the brains of AD patients with different Braak stages. As all samples were obtained from individuals older than 65 years, we next aimed to study whether this phenotype is age-dependent. To study this question in a human 3D model, we differentiated iPS cells to cerebral organoids (Fig. 4G). In contrast to the aged human brain, immunofluorescence analysis demonstrated no co-localization of APP-CT50 with PML in cerebral organoid sections.

### Reduction of PML bodies occurs in human hippocampal brain areas with high plaque load

**Fig. 5:**
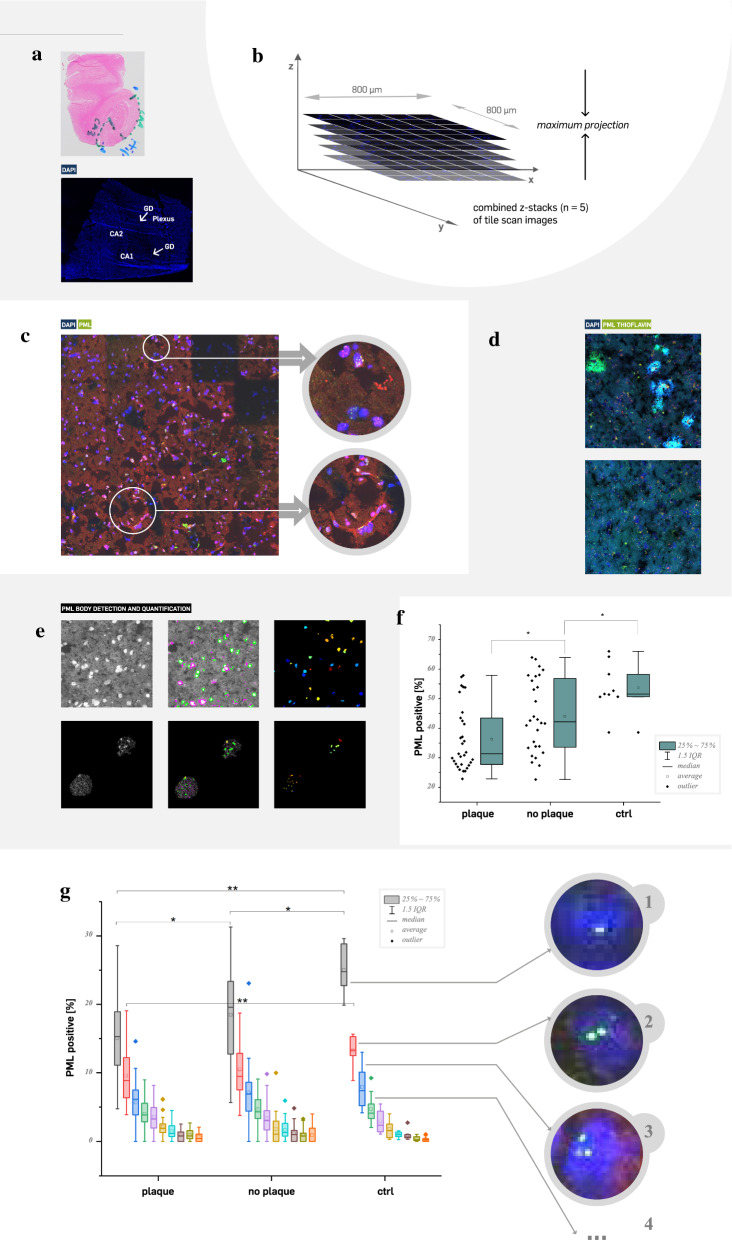
Significant enrichment of PML bodies in human brain areas with high plaque load. (**A**) Haemotoxylin eosin (HE) staining of human hippocampal sections was used to define Cornu Ammonis (CA) 1–3, Gyrus dentatus (GD), and Plexus areal. Parallel sections were used for PML immunofluorescence staining. DAPI co-staining was used for low-resolution tile-scan imaging and allocation of specific CA areas according to the initial HE staining. (**B**) Hippocampal CA1 or CA3 areas were used for high-resolution tile scan imaging. A single scan included 5 × 5 images with 5 z-stacks, which were subsequently combined using maximum projection. (**C**) A representative high-resolution (100 × objective) confocal tile-scan image demonstrates identification of PML bodies in DAPI counter-stained nuclei. (**D**) Tile-scan imaging was then established with Thioflavin co-staining to identify areas with high plaque load in the human hippocampus (CA1 or CA3). (**E**) CellProfiler™ software was used to automatically annotate and extract nuclei (DAPI channel in grey scale) from the image (upper row). Subsequently, PML body identification and quantification was done in the extracted nuclei. (**F**) All nuclei containing 1 to 5 PML bodies were used to determine the percentage of PML positive nuclei (*y* axis). Hippocampal areas with high plaque load (plaque, average = 36.2%) revealed a significant lower percentage of PML positive nuclei compared to areas without plaque (no plaque, average = 44.1%) (*p* < 0.05; every dot (left to each bar) indicates a single tile-scan experiment; for areas with high plaque load 31 tile-scans were analysed and quantified, 28 for no plaque, 9 for control). Tile-scans of control sections (individuals without any plaque pathology, average = 53.8%) revealed again higher percentage of PML positive nuclei compared to “no plaque” areas (*p* < 0.05). (**G**) Detailed analysis of cells containing one up to ten PML bodies per nucleus revealed significant differences. Nuclei with a single PML body were underrepresented (*p* < 0.05) in areas with high plaque load versus areas without plaques. Difference to control tile scans were highly significant (*p* < 10^−5^). In addition, nuclei with two PML bodies (red bars) also revealed highly significant differences to the control. Representative images for nuclei containing one, two, or three PML bodies are given

In order to study a potential pathophysiological relevance, we examined human brain tissue in more detail (Fig. 5). As the human hippocampus belongs to those brain areas revealing early pathological AD features, we studied the extent of PML bodies in the Cornu Ammonis areas 1 or 3 (CA1, CA3). CA regions were not evident for all human brain samples due to different quality (different post-mortem times, preparation artefacts), thus we limited our analysis to CA1 or CA3 areas, which were distinctly assignable, e.g. Figure 5A corresponds to a sample with CA1 assignment, but not CA3. Haematoxylin Eosin (HE) staining of human hippocampal frozen sections (samples from 15 AD individuals with different Braak stages) was used to identify the specific areas. Parallel sections (from the same individual) were used for low-resolution tile-scale imaging (DAPI channel, Fig. 5A) to allocate hippocampal areas according to HE staining. For subsequent detailed analysis, confocal tile-scan imaging was used including 5 × 5 tiles and 5 z-stacks (Fig. 5B). Afterwards, maximum projection algorithm was applied resulting in high-resolution imaging enabling identification of the number of PML bodies in each nucleus over an area of 800 × 800 µm within CA1 or CA3 (Fig. 5C). This imaging pipeline was further extended to identify areas of high plaque load (within CA1 or CA3) using Thioflavin co-staining (Fig. 5D). In total, we scanned 68 areas using this approach. All tile-scans were subsequently processed using CellProfiler™ software in order to identify and extract nuclei (Fig. 5E, upper row) and to detect and count the number of PML bodies within the extracted nuclei (Fig. 5E, bottom row). Data analysis of all cells containing between 1 and 5 PML bodies compared to all cells counted (cells without any PML body, cells with more than 5 PML bodies), revealed a significant (*p* < 0.05) reduced percentage of PML positive nuclei in areas with high plaque load (Fig. 5F, plaque) compared to plaque free areas (Fig. 5F, no plaque). Tile-scans from individuals without any neurodegenerative pathology (controls) demonstrated higher percentage of PML positive nuclei compared to the “no plaque” group (*p* < 0.05). More detailed analyses demonstrated that the observed significance is particularly caused by cells containing only one or two PML bodies per nucleus (Fig. 5G, *p < 0.05, **p < 10^−5^).

## Discussion

The amyloid precursor protein is a ubiquitously expressed protein, thus it is not surprising that many different cell types are capable to induce APP-CT (APP-CT50) signalling and to set up nuclear aggregates. FE65 was initially reported to be brain tissue-specific, and indeed, expression analysis suggested FE65 to be a neuronal protein [24–26]. TIP60 is also ubiquitously expressed with the highest amounts in testis and placenta. Thus, we conclude that APP-CT50/FE65/TIP60 signalling is a ubiquitous pathway with a preference in neuronal cells due to neuron-specific FE65 expression. Notably, APP-CT50 dissociation and nuclear translocation was described to predominantly occur through the amyloidogenic processing pathway [27]. This hypothesis is further supported by findings in APP-CT over-expressing mice revealing neuronal network abnormalities [28]. APP-CT has an impact on gene expression [9–12], and our findings of positive histone 3 K9 acetylation in the core of the APP-CT aggregates further support this hypothesis. The donut-like structure of the nuclear aggregates fits to this function as well, assuming that DNA is incorporated in (or associated to) the spherical aggregates as already shown for cells in G2 phase [29]. Thus, APP-CT50 aggregates might correspond to DNA incubation containers possibly modifying DNA in a way to change expression.

In order to better understand the nuclear APP-CT50-depending aggregates, we studied their composition and identified the two tumour suppressor proteins p53 and PML as additional components. According to our results in HEK293 cells, this interplay is strongly driven by the APP-CT50 nuclear translocation, as pure PML expression revealed a rather homogenous cellular distribution. The complex generation follows a temporally organized scheme with generation of small aggregates at an early phase and few large nuclear complexes at later time points. As these aggregates were evident in the human brain of aged patients but not in cerebral organoids, we conclude that the APP-CT50 nuclear signalling is age-dependent and potentially of relevance for the pathophysiology of Alzheimer’s disease. Staining of APP-CT50 and FE65 was only successful using a sophisticated protocol with defined order of antibody incubation, meaning that incubation with the secondary antibody for PML detection precluded the identification of APP-CT50 or FE65 in the complex. In contrast, usage of the secondary antibody as the last step (after application of the Avidin-TRITC complex to identify APP-CT50 of FE65) was successful to reveal co-localization. Thus, accessibility of the epitopes of APP-CT50 or FE65 was presumably masked by the secondary antibody used for PML staining, suggesting a localization of APP-CT50 and FE65 within PML spherical aggregates, which is in good agreement to other reports [29].

Relevance of APP-CT50/FE65/PML aggregates for the pathophysiology of AD was finally demonstrated by tile-scan imaging of hippocampal CA1 and CA3 areas. Our analysis revealed highly significant results demonstrating a reduction in the number of PML bodies in nuclei close to AD relevant hot spots with high plaque load. Assuming that these areas also correspond to elevated APP cleavage, we conclude that APP nuclear signalling involving the adapter protein FE65 is correlated to AD pathology. Expression of APP-CT50, FE65, TIP60 and PML in HEK293T cells caused a time-dependent fusion of the nuclear aggregates. Thus, APP-driven fusion of PML aggregates may occur in the AD brain in a similar fashion. Certainly, further studies are pivotal to understand the consequences of APP to PML body generation, fusion, and, in particular, their impact on neurodegeneration and dependence on different Braak stages.

## Conclusion

APP-CT50 signal transduction is of high relevance for AD and causes a reduction in the number of PML bodies in nuclei close to AD relevant hot spots. The composition of the nuclear aggregates includes APP-CT50, and tumor suppressor proteins PML and p53. Aggregates are associated to gene expression changes with putative impact in neurodegeneration.

## Supplementary Information

**Additional file1.** This file contains supplementary Figures and legends S1–S7.

**Additional file2.** This file contains a supplementary movie to Figure 4 A.

## Abbreviations

AD: Alzheimer’s disease
APP-CT: Amyloid precursor protein c-terminal domain
CA: Cornu ammonis
CMLE: Classic Maximum Likelihood Estimation
CO: Cerebral organoids
FITC: Fluorescein isothiocyanate
GFP: Green fluorescent protein
HE: Haematoxylin Eosin
HyD: Hybrid detector
iPS: Induced pluripotent stem (cell)
LDA: Linear discrimination analysis
PBS: Phosphate buffered saline
PML: Promyelocytic leukemia protein
PVDF: Polyvinylidene difluoride
ROI: Region of interest
STED: Stimulated emission depletion
TRITC: Tetramethylrhodamine

## Declarations

### Availability of supporting data

In case of acceptance, supporting data can be accessed at the Cell image library (http://www.cellimagelibrary.org/home).
