# Supplementary material for: Amyloid precursor protein elevates fusion of promyelocytic leukemia nuclear bodies in human hippocampal areas with high plaque load
Source: Acta Neuropathol Commun. 2021 Apr 13;9:66. doi: 10.1186/s40478-021-01174-x (PMC8042982; doi:10.1186/s40478-021-01174-x)
Supplement: Supplementary file 1 — Additional file1. This file contains supplementary Figures and legends S1–S7. [file 40478_2021_1174_MOESM1_ESM.docx]

**Supplement**

**Figure S1**

Figure S1 - HEK293 cells transfected with FE65-EGFP/TIP60 (w/o fluorophore) reveal a nuclear dot like phenotype. Cells with many tiny dots (red arrow) can be distinguished from cells with giant ring-like structures (yellow arrows).

**Figure S2**

Figure S2 - Electron microscopy imaging of HEK293 cells, transfected with FE65-EGFP/TIP60 (w/o fluorophore) revealed a circular phenotype of the nuclear aggregates. However, membrane-like structured were not evident.

**Figure S3**

Figure S3 - CellMask stain (purple) in FE65-EGFP (green), TIP60 (w/o fluorophore) transfected HEK293 cells failed to reveal a prominent membrane staining encapsulating the nuclear aggregates (arrow).

**Figure S4**

Figure S4 - STED imaging of FE65-EGFP, TIP60 (w/o fluorophore), PML-GFP (isoform IV) transfected cells. Upon fixation, immunofluorescence staining using anti histone3-K9ac antibody (red) was performed demonstrating active gene expression in some of the nuclear aggregates. Please note that the focus of this experiment was to identify acetylated histone within the nuclear aggregates. Thus, we spared to exclude GFP from the PML vector and transfected to GFP-labeled constructs.

**Figure S5**

A

B

Figure S5 - Additional experiment to IP given in Figure 2K. For details reader is referred to Legend of Figure 2.

**Figure S6**

A

B

Figure S6 – APP-CT/PML co-staining in the human brain. (A) Additional images (to Figure 3E) revealing PML/APP-CT co-localization in human brain sections. (B) For negative control, the primary APP-CT antibody was omitted revealing no co-localization of the red (background) signal to PML in green.

**Figure S7**

Figure S7 – Overview of plasmids used in this study. The coding protein-tags and fluorophores are shown corresponding to their N🡪C structure.
